# A Nurr1 Agonist Causes Neuroprotection in a Parkinson’s Disease Lesion Model Primed with the Toll-Like Receptor 3 dsRNA Inflammatory Stimulant Poly(I:C)

**DOI:** 10.1371/journal.pone.0121072

**Published:** 2015-03-27

**Authors:** Gaynor A. Smith, Emily M. Rocha, Thomas Rooney, Pascal Barneoud, Jesse R. McLean, Jonathan Beagan, Teresia Osborn, Madeleine Coimbra, Yongyi Luo, Penelope J. Hallett, Ole Isacson

**Affiliations:** 1 Neuroregeneration Research Institute, Harvard Medical School/McLean Hospital, Belmont, Massachusetts, United States of America; 2 Sanofi, Neurodegeneration and Pain Unit, Chilly-Mazarin, France; 3 Sanofi, Disposition, Safety & Animal Research, Department 1, Chilly-Mazarin, France; 4 Sanofi-Genzyme, Drug Metabolism and Pharmacokinetics Department, Waltham, Massachusetts, United States of America; Emory University, UNITED STATES

## Abstract

Dopaminergic neurons in the substantia nigra *pars compacta* (SNpc) are characterized by the expression of genes required for dopamine synthesis, handling and reuptake and the expression of these genes is largely controlled by nuclear receptor related 1 (Nurr1). Nurr1 is also expressed in astrocytes and microglia where it functions to mitigate the release of proinflammatory cytokines and neurotoxic factors. Given that Parkinson’s disease (PD) pathogenesis has been linked to both loss of Nurr1 expression in the SNpc and inflammation, increasing levels of Nurr1 maybe a promising therapeutic strategy. In this study a novel Nurr1 agonist, SA00025, was tested for both its efficiency to induce the transcription of dopaminergic target genes *in vivo* and prevent dopaminergic neuron degeneration in an inflammation exacerbated 6-OHDA-lesion model of PD. SA00025 (30mg/kg p.o.) entered the brain and modulated the expression of the dopaminergic phenotype genes TH, VMAT, DAT, AADC and the GDNF receptor gene c-Ret in the SN of naive rats. Daily gavage treatment with SA00025 (30mg/kg) for 32 days also induced partial neuroprotection of dopaminergic neurons and fibers in rats administered a priming injection of polyinosinic-polycytidylic acid (poly(I:C) and subsequent injection of 6-OHDA. The neuroprotective effects of SA00025 in this dopamine neuron degeneration model were associated with changes in microglial morphology indicative of a resting state and a decrease in microglial specific IBA-1 staining intensity in the SNpc. Astrocyte specific GFAP staining intensity and IL-6 levels were also reduced. We conclude that Nurr1 agonist treatment causes neuroprotective and anti-inflammatory effects in an inflammation exacerbated 6-OHDA lesion model of PD.

## Introduction

Dopaminergic neurons in the substantia nigra *pars compacta* (SNpc) are characterized by the expression of specific genes required for dopamine regulation and the expression of these genes is controlled by the nuclear receptor related 1 (Nurr1) protein [1,2,3]. Abolishing Nurr1 causes the loss of this essential dopaminergic phenotype *in vitro* [4] and *in vivo* [5]. Specifically, Nurr1 is required for the transcription of tyrosine hydroxylase (TH), vesicular monoamine transporter (VMAT), dopamine active transporter (DAT) and aromatic l-amino acid decarboxylase (AADC) and hence plays a fundamental role in both the differentiation of dopaminergic neurons in embryonic stages and the long-term maintenance of the dopaminergic phenotype throughout life [1,2,3]. Polymorphisms in the NURR1 gene have been identified in patients with both sporadic and familial forms of Parkinson’s disease (PD) [6,7,8,9], however the prevalence of these mutations is rare. Although the role of wild-type Nurr1 in the onset and progression of PD is not well established, there is a reduced expression in the SNpc with increased age [10], which is the major risk factor in the development of PD. Reduced Nurr1 expression is also observed in PD patients compared to age matched controls [11,12]. Nurr1 (+/-) knock out mice and tamoxifen inducible Nurr1 (-/-) knock out mice display a dopamine neuronal phenotype [13], a reduction of dopamine release [13] and show progressive behavioral deficits [14], whereas embryonic Nurr1 (-/-) knock out mice die by post natal day two [15]. Conversely, overexpressing Nurr1 using a viral vector rescues a-synuclein mediated degeneration of dopaminergic neurons by restoring GDNF levels [16]. Nurr1 also causes the induction of brain-derived neurotrophic factor (BDNF) transcription [17]. Therefore neurotrophic factors may also act synergistically to promote dopaminergic neuron survival.

In addition, Nurr1 negatively regulates inflammation [18], which is also considered a risk factor for the development of PD [19]. We have previously shown that toll-like receptor 4 (TLR4) stimulation by lipopolysaccharide (LPS) or activation of TLR3 by polyinosinic-polycytidylic acid (poly(I:C)) primes dopaminergic neurons for a subsequent oxidative stress insult, caused by 6-hydroxydopamine (6-OHDA) [20,21]. This results in exacerbated midbrain dopamine neuron degeneration compared to 6-OHDA given alone [20,21]. Administration of an interleukin-1 receptor antagonist in this model resulted in significant reductions of the pro-inflammatory cytokines TNFα and IFNγ and attenuated the loss of dopamine neurons [21]. We have previously found that a low dose of 6-OHDA does not cause major midbrain dopamine degeneration without prior inflammatory stimulant priming [20, 21]. A single low dose injection of poly(I:C) or LPS induces the expression of inflammatory markers at 7–12 days post injection in the SNpc, which then subsides [20, 21]. In a proof of concept study, at that time-point retrograde degeneration of the vulnerable dopamine neurons (caused by low-dose intrastriatal 6-OHDA-degeneration) was almost doubled by this prior TLR-induced cytokine peak in the substantia nigra [21]. Following inflammatory stimulation on TLRs, Nurr1 acts as a transcription factor for the removal of NF-kB from the promoter regions of proinflammatory cytokine genes, which would otherwise cause their transcription [18]. The suppression of damaging reactive oxygen species signals from astrocytes and microglia is also negatively regulated by Nurr1 levels [18]. Nurr1 knock down in microglia and astrocytes of mice exacerbates dopaminergic neuron degeneration in the SN caused by an LPS injection [18].

Therefore given Nurr1’s role in both dopaminergic neuronal phenotype maintenance and in mitigating pro-inflammatory signals, increasing Nurr1 levels or activating Nurr1 may be a promising strategy for the treatment of PD [22]. In the following we describe the neuroprotective and anti-inflammatory effect of a novel Nurr1 agonist compound in an inflammation exacerbated 6-OHDA lesion model of PD.

## Materials and Methods

### Animals

Female Sprague-Dawley rats weighing ~250 g (Charles River Laboratories) were housed in standard conditions with ad libitum access to food and water under a 12/12 hour light/dark cycle. This work was approved by the McLean Hospital IACUC under protocol 13-6/2-14.

### Nurr1 agonist treatment paradigms

The Nurr1 agonist (SA00025) (reference: A.G. Almario, P. Lardennois, A. Olivier, PCT Int. Appl. (2008), WO2008034974A1) was received from Sanofi. On each day testing a 30mg/kg working solution of SA00025 was made fresh by dissolving SA00025 in 0.6% methylcellulose and 0.5% Tween-80 in distilled water. 0.6% methylcellulose and 0.5% Tween-80 in distilled water was used as the vehicle. In the first experimental paradigm naive rats were gavaged daily for 7 consecutive days. In this paradigm rats were killed at 1, 4, 12 or 24 hrs after the last gavage (N = 3–4/group). In the second experimental paradigm, treatment was started 1 day post intra-nigral poly I:C injection (day 1) and was administered daily for the duration of the experiment (32 days), including during the day rats received intra-striatal 6-OHDA (day 12). Rats were sacrificed 24 hrs after the final administration of SA00025 (day 33), (N = 8/group).

### PK analysis

Rats were terminally anesthetized and perfused transcardially with heparinized saline (0.1% heparin in 0.9% saline) and whole brains were removed and weighed. Brains were homogenized in distilled water at a volume (μl) that was 2x brain weight. A 5μL aliquot of sample was injected onto a Phenomenex Luna C8 (50x2.0 mm) 5 mm HPLC column with a Shimadzu SILHTC auto sampler and an integrated HPLC pumping system Shimadzu LC10AD. The compound was detected by an Sciex API 5000 Mass Spectrometer with a positive ESI ionization mode. Mobile phase A was 95% acetonitrile in water, mobile phase B was 10 mM ammonium acetate buffer in water, pH 7.0 with a flow rate of 500 μL/min. The starting condition for HPLC gradient was 25:75 (A/B) at time 0 min, 100:0 (A/B) from 0.1 to 1.9 min and 25:75 (A/B) from 2 to 3 min. Multiple reaction monitoring (MRM) was used to monitor the compound with m/z transitions 363.2–347.2 and a retention time of 1.25 min.

### qPCR

For gene expression analysis, the RNA was extracted from dissected SN tissue samples using RNeasy Mini spin kit and shredder columns (Qiagen, USA) according to manufacturers instructions. 85–150 ng of RNA was used for cDNA preparation using Superscript III First-Strand Synthesis System (Invitrogen) according to manufacturers instructions. Quantitative qPCR was performed with SYBER Green Master Mix and qPCR primers (Taqman premade and validated primers for TH, VMAT, DAT, c-RET and Nurr1). The expression of the gene of interest was determined in triplicate samples for the cDNA preparation made for each rat SN sample. The Ct value of each target gene was normalized against the Ct value of the reference gene (Ct(target)-Ct(GAPDH)). Results were analyzed using the ΔΔCt method (ΔCt(Nurr1 agonist treated)-ΔCt(vehicle treated control)). The relative expression caused by the Nurr1 agonist compound was calculated by 2^-ΔΔCt and represented as fold change compared to compared to vehicle treatment (= 1).

### Western Blot analysis

Rats were terminally anesthetized and perfused transcardially with heparinized saline (0.1% heparin in 0.9% saline) and brains were cut in serial sections to 1mm. The SN was dissected from each rat and each suspended in ice cold buffer containing: 300 mM sucrose in TE buffer (Bio-Rad). Phosphatase inhibitors I and II (1:100), proteinase inhibitors (1:100) (Thermo Halt proteinase inhibitor single use cocktail) and EDTA (Thermo) (1:100) were then added. Tissues were homogenized for 15 secs and sonicated with three 1 second pulses. 30mg protein homogenates, determined by a BSA assay (Pierce), were loaded on to each gel, using the Criterion precast 4–12.5% SDS polyacrylamide gel system (Bio-Rad). The proteins were then transferred to a PVDF membrane at 21 V and 2.5 amps. Membranes were washed in Tris-buffered saline with 0.1% Tween 20 (TBS-T) and then blocked in 5% protein blocker (Bio-Rad). The membrane was then incubated overnight at 4°C in anti-TH (Pel-Freez 1:1000) and anti-GAPDH (Millipore, 1:5000). HRP-conjugated secondary antibodies were applied to the membranes for 1 h at room temperature. The blot was treated with ECL-Plus (Amersham Biosciences) and exposed using ChemiDoc XRS and image Lab software to visualize the bands. Optical density analysis (NIH image) was used to determine the relative abundance of TH compared to GAPDH levels.

### Stereotaxic delivery of polyI:C and 6-OHDA

Stereotaxic surgery and polyI:C/ 6-OHDA solution preparation was performed according to [20]. In brief, rats were first anesthetized with ketamine and xylazine (60 mg/kg and 3 mg/kg respectively, i.p.). A total of 20 μg of poly(I:C) (Axxora), in a volume of 2μl, was then delivered to the SN by stereotaxic injection at a rate of 0.5 μl/min using microinfusion pumps (Stoelting Co, Wood Dale, IL) with a 5 minute wait time after injection. The stereotaxic coordinates used for the intra-nigral injection of poly(I:C) were: AP:− 5.5 mm, ML: − 2.0 mm, and DV: 7.5 mm relative to bregma. Following surgery rats were returned to home cages for 11 days before being re-anesthetized for intra-striatal 6-OHDA delivery. Rats were injected with 5μg of 6-OHDA (Sigma-Aldrich, St Louis, MO), prepared as free base, in a single 3.5 μl deposit at a rate of 0.5 μl/ min. A 5 min wait time was allowed after injection. Striatal injection coordinates were as follows: AP: +0.2, ML: − 3.0, DV: − 5.0 relative to bregma.

### Histology

Rats were terminally anaesthetized with sodium pentobarbital and perfused transcardially with 25 ml of heparinised saline (0.1%) followed by 100 ml of 4% paraformaldehyde in phosphate buffer. Brains were post-fixed in 4% paraformaldehyde for 24 h before placing them in 30% sucrose. Coronal sections were cut to 40 μm, and stored in antifreeze at -20°C until use. For immunohistochemistry, sections were washed in PBS followed by 3% hydrogen peroxide for 7 min to quench endogenous peroxidases. After three washes in PBS, the sections were incubated in 5% normal goat serum in 0.1% Triton X-100 in PBS for 1 hour before the following primary antibodies were added: Anti-IBA1 (Wako, 1:200), anti-NeuN (Millipore, 1:1000) or anti-TH (Pel-Freez 1:500). Primary antibodies were left at 4°C overnight. After washing in PBS, sections were incubated in goat anti-mouse/ rabbit biotinylated secondary antibody (Vector Laboratories 1:200). Staining of the tissue-bound antibody was visualized using a standard peroxidase-based method using an ABC kit (Vector Laboratories) and chromogen, 3,3′-diaminobenzadine (DAB, Sigma). NeuN and TH co-stains were done sequentially and nicklel added to the DAB reaction for anti-NeuN peroxidase reaction. Following immunohistochemistry tissue sections were mounted onto slides, dehydrated and coverslipped.

For co-immunofluorescence stains, tissue sections were incubated in blocking solution with anti-IBA1 (Wako, 1:200) and anti-GFAP (Chemicon, 1:1000) at 4°C and left overnight. Sections were washed in 3x in PBS and incubated with Alexa Fluor secondary antibodies (Life Technologies, 1:200) in PBS and applied for 2 hrs at room temperature. Sections were washed in 3x in PBS and mounted on slides. Coverslips were added using aqueous mounting media.

### Multiplex ELISA

Tissue samples were collected and suspended in PBS, with the addition of phosphatase inhibitors I-II (1:100) and protease inhibitors (1:100), (Thermo Halt proteinase inhibitor single use cocktail). Samples were homogenized and a portion of the supernatant was reserved for protein determination (BCA Assay, Pierce). Samples were then analyzed for the simultaneous detection of IL10, IL6, IL1a, MCP1, MIP1a, MIP2, MIP3a, RANTES, TNFa, Fractalkine, ILb, IL-2, MDC and TGFb1 using a multiplex ELISA based format for rat protein detection, performed in triplicate. Testing was performed independently through Aushon BioSystems using the Cira immunoassay platform and the relative amounts of proteins determined for Nurr1 agonist and vehicle treated rat tissue in pg/mg.

### Histological quantification and statistics

Estimates of the number of remaining dopamine neurons in the SNpc were quantified on sections using Stereo Investigator (MBF Bioscience), by an investigator bind to the treatment conditions. Dopamine neurons that were TH and NeuN positive were quantified, on both the contralateral and ipsilateral sides, using the Optical Fractionater Workflow function at the following defined parameters: number of sections (4–5/ rat), section evaluation interval 6 and the measured section thickness (~18–25μm/ section) at 20x magnification. Guard zones were set to 4μm. Counts were presented as a% of the contralateral side.

## Results

### SA00025 entered the brain and modulated the transcription of dopaminergic target genes

**Fig 1:**
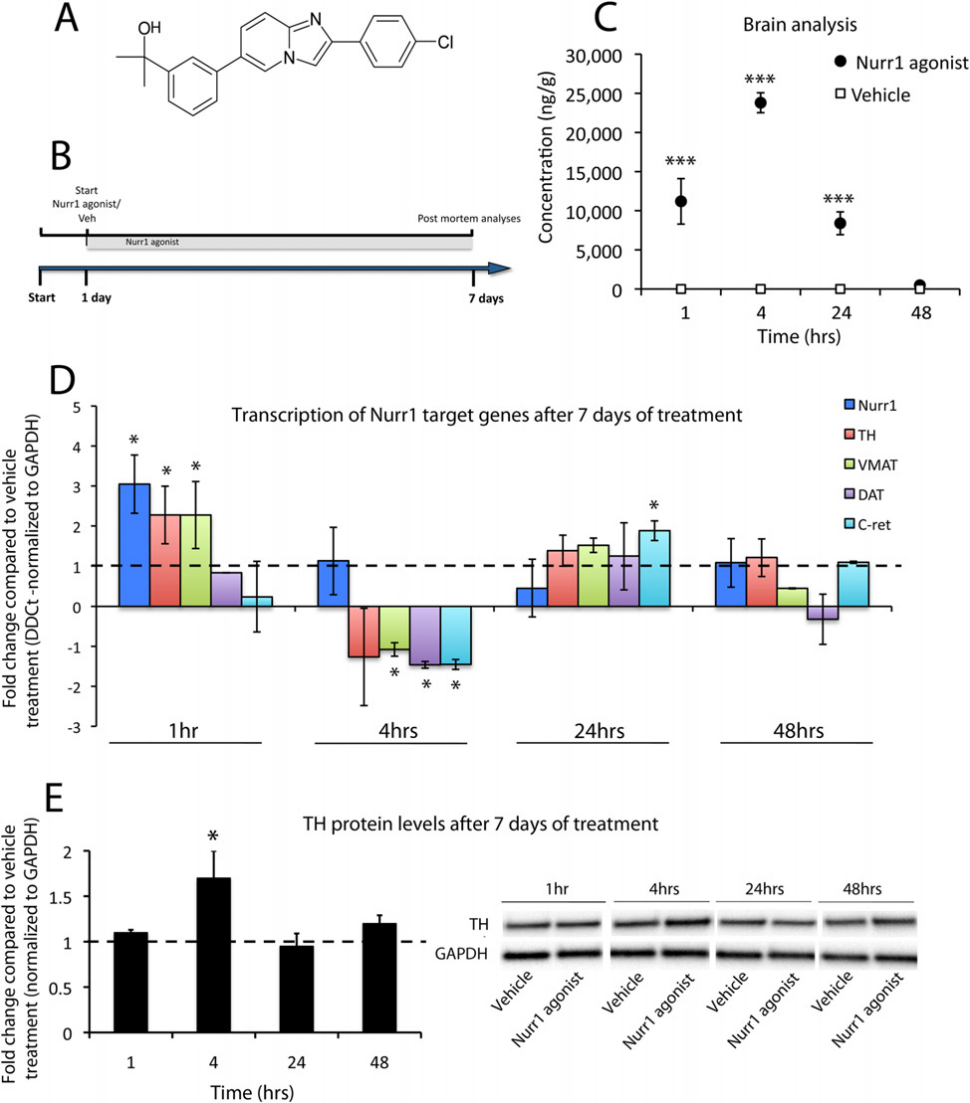
SA00025 entered the brain and modulated the transcription of dopaminergic target genes. The chemical structure of SA00025: 2-{3-[2-(4-chlorophenyl)imidazo[1,2-a]-pyridin-6-yl]phenyl}propan-2-ol (A). The Nurr1 agonist compound was administered once daily for 7 consecutive days (30mg/kg/ day) in naive rats by oral gavage (B). The compound was observed in the brain at 1 hour after the last gavage and reached a maximum concentration at 4 hrs post-gavage treatment (C). High levels of the compound were still observed in the brains of rats at 24 hrs and returned to baseline at 48 hrs (C). SA00025 caused a transcriptional upregulation of Nurr1, TH and VMAT at 1-hour post daily gavage and a transcriptional downregulation of VMAT, DAT and c-RET at 4 hours post-gavage treatment (D). A normalization of Nurr1, TH and VMAT expression was observed at 4–48 hours post-gavage treatment (D). Transcription of all dopaminergic target genes was equivalent between compound and vehicle treated rats at 48 hours post-gavage treatment (D). TH protein levels were elevated at 4 hours post-gavage treatment (E). Significance is annotated as p<0.05* & p<0.001*** compared vehicle treatment (dashed lines), unpaired *T*-tests and ANOVA. N = 4–8/ group. Graphs are expressed as mean ± SEM.

The structure of the small molecule Nurr1 agonist used in this study is shown in Fig. 1A. SA00025 is a potent agonist of Nurr1 (EC50 2.5nM) in HEK293 cells transfected with full length human Nurr1. The agonist was not found to activate panel of 40 other nuclear receptors, including RXR (manuscript in preparation). SA00025 was given by daily oral administration of 30mg/kg for 7 days (Fig. 1B). Following 7 days of daily oral treatment, PK analysis or post mortem showed that SA00025 entered the brain and confirmed elevated brain exposure at 1, 4 and 24 hrs after the last administration (F_3,18_ = 31.22, p<0.001). The compound was present in brain homogenates at 1-hour after the final oral treatment and reached a maximum concentration at 4 hours (Fig. 1C). SA00025 treatment significantly modulated the expression of Nurr1 and dopaminergic target genes from 1–48 hrs after oral administration for 7 days (F_3,18_ = 5.61, p<0.05). Specifically, SA00025 induced a transcriptional upregulation of Nurr1, TH and VMAT at 1-hour post daily oral treatment and a transcriptional downregulation of VMAT, DAT and c-RET at 4 hours post Nurr1 agonist treatment (Fig. 1D). A normalization of Nurr1, TH and VMAT mRNA expression was observed at 4 hours post oral administration and an increase in c-Ret observed at 24 hrs (Fig. 1D). Concomitantly, protein levels of TH were significantly elevated at 4 hours following Nurr1 agonist treatment (F_3,18_ = 3.22, p<0.05) compared to vehicle treatment (Fig. 1E).

### SA00025 caused neuroprotection in the 6-OHDA lesion model primed with the TLR3 dsRNA inflammatory stimulant (poly(I:C))

**Fig 2:**
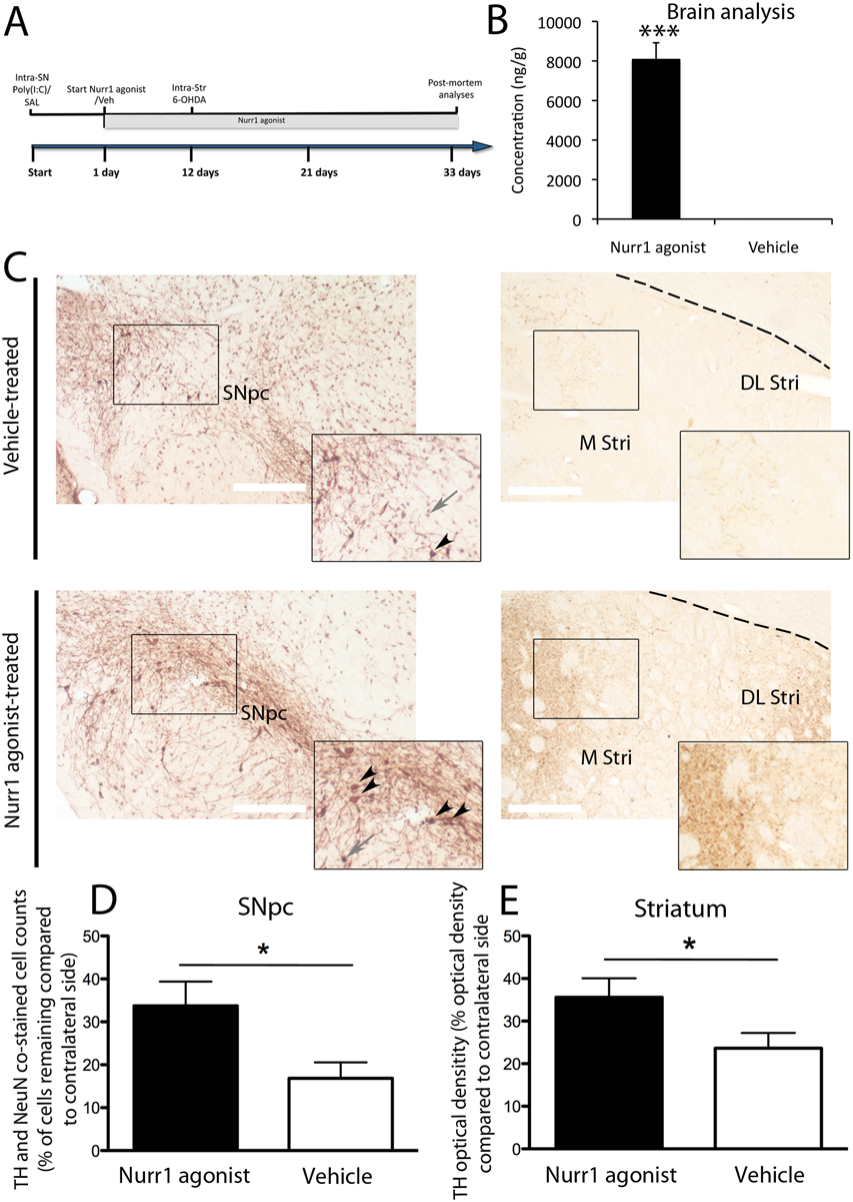
SA00025 caused neuroprotection in the 6-OHDA lesion model primed with dsRNA inflammatory stimulant (poly(I:C)). Rats received an initial unilateral injection of poly(I:C) in the SN (Start—day 0) and a subsequent unilateral injection of 6-OHDA in the striatum (day 12) (A). Rats were also administered daily with the Nurr1 agonist (30mg/kg/ day) or vehicle treatment throughout the paradigm (day 1–32) and were sacrificed at day 33 (A). 24 hrs following the last gavage treatment levels of the compound were significantly elevated in brain homogenates and was absent in vehicle treated conditions (B). Representative photomicrographs indicate the Nurr1 agonist caused a protective affect on TH +ve (brown) and NeuN +ve (grey) neurons within the SNpc and TH +ve fibers in the striatum (C). Rats had a significant sparing of dopaminergic neurons in the SNpc after 32 days of treatment with the compound, compared to vehicle treatment (D). Dopamine neuron fibers in the rostral striatum were also significantly spared with Nurr1 agonist treatment compared to vehicle treatment (E). Significance is annotated as p<0.05*, unpaired *T*-tests. N = 6–8/ group. Graphs are expressed at mean ± SEM. Abbreviations: dorsal lateral striatum (DL Stri) & medial striatum (M Stri). Scale bars = 200μm. TH and NeuN +ve neurons are indicated by black arrow heads and NeuN +ve neurons are indicated by grey arrows.

The neuroprotective and anti-inflammatory effect of SA00025 was tested in a model of viral-like inflammation exacerbated by oxidative damage from 6-OHDA [20]. In this model [20] rats first receive an initial unilateral injection of poly(I:C) in the SN (Start—day 0) and then after 12 days a second unilateral infusion of low dose 6-OHDA in the dopamine terminal region of the striatum (Fig. 2A). Rats were also administered the Nurr1 agonist or vehicle throughout the paradigm period (day 1–32) and were sacrificed at day 33 (Fig. 2A). Appropriate brain exposure of the compound was confirmed in this experiment with elevated brain levels measured 24h after treatment (T_1,16_ = 9.81, p<0.001), (Fig 2B). Our post-mortem analysis showed that the Nurr1 agonist compound was neuroprotective on TH and NeuN positive neurons within the SNpc, using unbiased stereology, and also preserved TH positive fibers in the striatum, as observed by densitometry measurements (Fig. 2C). Rats had a significant sparing of dopaminergic neurons in the SNpc after 32 days of treatment with the Nurr1 agonist compound (T_1,16_ = 2.16, p<0.05), compared to vehicle conditions (Fig. 2D). There was no significant difference between the number of TH and NeuN positive dopamine neurons between Nurr1 agonist and vehicle treatment conditions on the contralateral side (vehicle = 7441 ± 491.6; SA00025 = 7916 ± 408.1, T_1,16_ = 0.27, p = n.s), following injection with the TLR3 Stimulant polyI:C. The intensity of TH staining within individual dopaminergic cell bodies of Nurr1 treated rats was significantly higher compared to vehicle conditions on the ipsilateral side (vehicle = 36 ± 3.12; SA00025 = 47.6 ± 4.01, T_1,16_ = 2.18, p<0.05) and the contralateral side (vehicle = 38.15 ± 2.11; SA00025 = 47.1 ± 3.21, T_1,16_ = 2.33, p<0.05). Dopamine neuron fibers in the rostral striatum were also significantly spared with administration of the compound (T_1,16_ = 1.86, p<0.05), compared to vehicle treatment (Fig. 2E).

### SA00025 displayed anti-inflammatory activity in the 6-OHDA lesion model primed with the TLR3 dsRNA inflammatory stimulant (poly(I:C))

**Fig 3:**
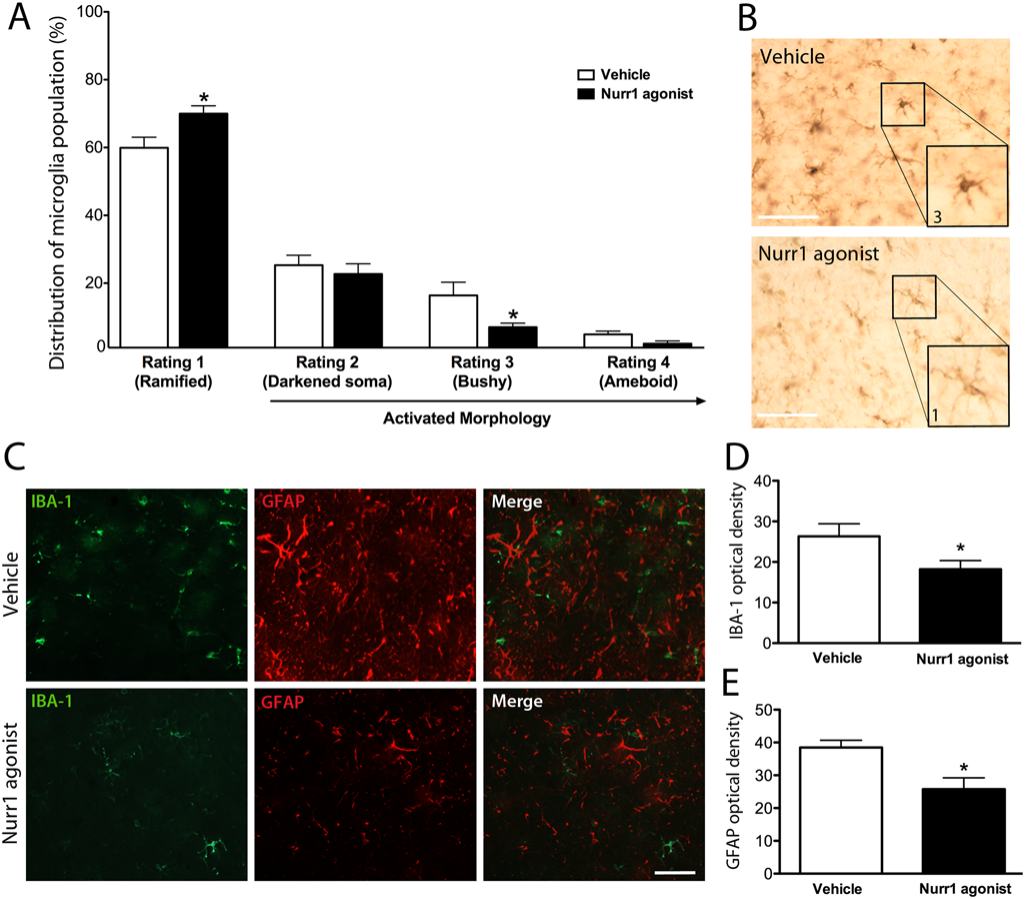
SA00025 induced anti-inflammatory activity in the 6-OHDA lesion model primed with dsRNA inflammatory stimulant (poly(I:C)). There was a significant increase in the amount IBA-1 +ve microglia in the SNpc scored as rating 1 (ramified and resting) and a significant decrease in microglia scored at rating 3 (bushy and reactive) with 32 days of Nurr1 agonist treatment, compared to vehicle (A). Representative photomicrographs show that Nurr1 agonist treatment caused a reduction in IBA-1 +ve microglia that were bushy and reactive and an increase in IBA-1 +ve microglia that were ramified and resting in the SNpc (B). Representative photomicrographs show that Nurr1 agonist treatment also caused a reduction in the immunofluorescent intensity of IBA-1 +ve microglia (green) and GFAP +ve astrocytes (C). Optical density analysis indicated that Nurr1 agonist treatment caused a significant decrease in IBA-1 (D) and GFAP (E) staining in the SNpc, compared to vehicle conditions. Significance is annotated as p<0.05*, 2- way ANOVA. N = 6–8/ group. Graphs are expressed at mean ± SEM. Scale bars = 100μm.

At the end of Nurr1 agonist administration to rats that received an intra-nigral poly(I:C) injection followed by an intra-striatal 6-OHDA injection, we found that there was a significant morphological change of IBA-1 positive microglia in the SNpc. SA00025 treatment caused significantly more microglia residing in a resting state (ramified morphology) and a significant decrease in reactive microglia (bushy morphology) (F_3,40_ = 3.49, p<0.05) compared to vehicle treatment (Fig. 3A), depicted in (Fig. 3B). There was no significant difference in the total amount of microglia between vehicle and Nurr1 agonist treated groups (T_1,16_ = 0.98, p = n.s.). SA00025 treatment caused a reduction in the immunofluorescence intensity of both IBA-1 positive microglia and GFAP positive astrocytes in the SNpc compared to vehicle treatment (Fig. 3C). Optical density analysis indicated that SA00025 administration caused a significant decrease of both IBA-1 (T_1,16_ = 3.09, p<0.05) and GFAP (T_1,16_ = 3.86, p<0.05) immunofluorescence (Fig. 3D-E).

The protein levels of several key proinflammatory cytokines were then assessed in the SN of rats treated with the compound or vehicle. A significant reduction in IL-6 was observed with Nurr1 agonist treatment (T_1,16_ = 1.96, p<0.05), yet IL10, IL1a, MCP1, MIP1a, MIP2, MIP3a, RANTES, Fractalkine, TNFa, ILb, IL-2, MDC and TGFb1 remained unchanged (Table 1).

**Table 1:** Cytokine and Chemokine protein levels in the SNpc following Nurr1 agonist treatment.

Cytokines & chemokines ^a^	Poly(I:C) & 6-OHDA Vehicle treatment	Poly(I:C) & 6-OHDA Nurr1 agonist treatment
**IL10**	3.62 ± 0.84	2.17 ± 0.54
**IL1a**	1.40 ± 0.30	1.98 ± 0.19
**IL6**	144.59 ± 24.63	90.51 ± 21.94*↓ ^b^
**MCP1**	22.97 ± 3.65	20.20 ± 1.75
**MIP1a**	2.79 ± 0.40	2.62 ± 0.35
**MIP2**	0.52 ± 0.06	0.66 ± 0.13
**MIP3a**	3.89 ± 0.56	4.18 ± 0.57
**RANTES**	8.77 ± 0.52	8.03 ± 0.48
**TNFa**	11.93 ± 1.46	18.14 ± 6.12
**Fractalkine**	65.78 ± 4.50	62.44 ± 5.18
**IFNg**	106.76 ± 16.81	95.36 ± 9.26
**IL1b**	30.51 ± 4.69	29.84 ± 1.35
**IL2**	10.97 ± 2.16	9.01 ± 1.24
**MDC**	0.11 ± 0.01	0.11 ± 0.01
**TGFb1**	137.47 ± 35.98	123.31 ± 10.46

^a^ Cytokines and chemokine levels in the SN (pg/mg), analyzed by multiplex ELISA

^b^ Data is represented as mean ± SEM and significance is annotated as *p<0.05

## Discussion

The transcription of Nurr1 has fundamental roles to maintain the midbrain dopamine neuronal phenotype [1,2,23] and decrease the response to inflammatory insults [18] *in vivo*. In our model viral-like stimulation was induced using poly(I:C), causing an exacerbated response to subsequent oxidative stress [20]. It has been proposed that inflammogens, such as viruses, may play an etiological role in the development of PD and may exacerbate pathogenesis through the TLR signaling pathways [19]. Inflammation exacerbates dopamine neuron degeneration *in vivo* caused by a-synuclein overexpression [24], deficiencies in known PD risk genes [25,26] and pesticide exposure [27,28]. Therefore we have explored the therapeutic potential of a novel Nurr1 agonist, SA00025, compound to prevent the degeneration of dopamine neurons in an animal model of PD, primed with TLR3 activator poly(I:C).

We first confirmed that a single and chronic dosing of SA00025 was able to achieve significant brain levels with an extended half-life and it therefore has a favorable kinetic profile. Since physiologically active Nurr1 initiates and maintains the transcription of TH, VMAT, DAT and AADC [1,2], required for dopamine handling, synthesis and release, we then tested whether the Nurr1 agonist could modulate the transcription of these genes. Following a 7-day daily dosing treatment regime Nurr1, TH and VMAT were upregulated 1 hr after the last administration in the midbrain, confirming the action of the compound to induce Nurr1-dependant dopaminergic target gene transcription *in vivo*. At 4hrs TH protein levels were increased yet the transcription of TH was decreased. We suggest that the transcriptional repression of TH was caused by the increase in TH protein levels, possibly acting as a compensatory response to normalize TH protein levels to baseline. Nurr1, TH and VMAT were either downregulated or normalized at 4 hrs after the final administration. This likely occurs because of a tight regulatory system acting to compensate in response to the induced increase in protein levels. Nurr1 also controls the transcription of the GDNF receptor, c-Ret, and upregulation of this gene was seen at 24 hrs, indicating a slower transcriptional regulation than dopaminergic target genes. Nurr1 is an essential regulator for Ret expression in dopamine neurons [29,30] and increasing levels of c-ret may therefore enhance neurotrophic signaling [16] and TH mRNA levels [31].

We evaluated whether SA00025 caused neuroprotection in an oxidative stress rodent model of PD primed with polyI:C [20]. Using this model, we showed a protection of dopamine neurons in the SNpc and data to support that dopaminergic fibers were also spared in the striatum. The immunoreactivity of TH within dopamine cell bodies was also increased by SA00025 indicating that remaining dopamine neurons may also have a greater capacity for neurotransmitter synthesis. We have previously found that the inflammatory response initiated by the polyI:C injection induces a shift in microglial morphology that corresponds to a more reactive state and an increase in the proinflammatory cytokines and chemokines: IL-1b, IL-6, TNFa, MCP-1, RANTES and TGFb1 [20]. We have also previously shown that pro-inflammatory triggers were associated with enhanced dopaminergic degeneration [20,21]. In this paradigm, Nurr1 agonist treatment was associated with a decrease in reactive microglial morphology and a decrease in the optical density of GFAP positive astrocytes. At the time of post mortem, cytokine and chemokine analysis revealed that the Nurr1 agonist treatment could selectively reduce IL-6 in the SNpc. It is also possible that Nurr1 agonist treatment may alter the expression of other inflammation associated chemokines and cytokines at different time points post intracranial injections of polyI:C and 6-OHDA. IL-6 is secreted by macrophages and T-cells upon TLR3 stimulation [20,32,33,34,35], and is downstream of the Fas ligand pathway [36,37]. This pathway and IL-6 is enhanced in both PD patients [37,38] and in mice exposed to MPTP and 6-OHDA [39,40]. IL-6 has also been shown to be significantly elevated in cases of traumatic brain injury [41], Alzheimer’s disease [42] and Huntington’s disease [43]. This supports the idea that the long-term action of the Nurr1 agonist to decrease IL-6 may be beneficial in PD and other inflammatory related neurological disorders. The reduction of IL-6 by Nurr1 agonist treatment may occur because it promotes the removal of NF-kB from the promoter regions of proinflammatory cytokine genes [18]. Given this hypothesis it is also possible that the phosphorylation state of NF-kB may be altered and this warrants further investigation.

In addition to oxidative stress, genetic models of PD are also associated with inflammation. Reactive microglia morphological changes and proinflammatory cytokine expression are observed in the prodromal phases of the AAV-A53T a-synuclein induced neurodegeneration model [44], and isolated microglia cells from the transgenic mutant LRRK2 (R1441G) mouse model have a more reactive profile compared to wild-type cells [45]. Genetically obliterating LRRK2 *in vivo* causes neuroprotection against a-synuclein overexpression by suppressing the recruitment of chronically activated proinflammatory myeloid cells to the site of injury [46]. Therefore Nurr1 agonist treatment is also a candidate for the treatment of genetic forms of PD, including the most prevalent LRRK2 mutation causing form, and may help mitigate immune cell recruitment and decrease local inflammation.

## Conclusions

Based on these findings we conclude that Nurr1 agonist treatment warrants further investigation as a PD therapy. There may be several benefits of increasing Nurr1 activity. Firstly, since Nurr1 is downregulated in both normal aging [10] and in PD patients [11,12], Nurr1 agonist treatment may help to improve dopamine synthesis, handling and release during aging and after significant degeneration. Secondly, our data support an anti-inflammation role, which may be advantageous in the initial stages of PD, since neuronal degeneration may be triggered and exacerbated by inflammatory insults [19].

## References

[1] Alavian KN, Jeddi S, Naghipour SI, Nabili P, Licznerski P, Tierney TS. The lifelong maintenance of mesencephalic dopaminergic neurons by Nurr1 and engrailed. J Biomed Sci. 2014; 21: 27 10.1186/1423-0127-21-27 24685177 PMC3998737

[2] Jankovic J, Chen S, Le WD. The role of Nurr1 in the development of dopaminergic neurons and Parkinson's disease. Prog Neurobiol. 2005; 77: 128–138. 16243425 10.1016/j.pneurobio.2005.09.001

[3] Luo Y. The function and mechanisms of Nurr1 action in midbrain dopaminergic neurons, from development and maintenance to survival. Int Rev Neurobiol. 2012; 102: 1–22. 10.1016/B978-0-12-386986-9.00001-6 22748824

[4] Sonntag KC, Simantov R, Kim KS, Isacson O. Temporally induced Nurr1 can induce a non-neuronal dopaminergic cell type in embryonic stem cell differentiation. Eur J Neurosci. 2004; 19: 1141–1152. 15016073 10.1111/j.1460-9568.2004.03204.x PMC2614072

[5] Baffi JS, Palkovits M, Castillo SO, Mezey E, Nikodem VM. Differential expression of tyrosine hydroxylase in catecholaminergic neurons of neonatal wild-type and Nurr1-deficient mice. Neuroscience. 1999; 93: 631–642. 10465447 10.1016/s0306-4522(99)00124-4

[6] Tan EK, Chung H, Chandran VR, Tan C, Shen H, Yew K, et al Nurr1 mutational screen in Parkinson's disease. Mov Disord. 2004; 19: 1503–1505. 15390059 10.1002/mds.20246

[7] Grimes DA, Han F, Panisset M, Racacho L, Xiao F, Zou R, et al Translated mutation in the Nurr1 gene as a cause for Parkinson's disease. Mov Disord. 2006; 21: 906–909. 16532445 10.1002/mds.20820

[8] Hering R, Petrovic S, Mietz EM, Holzmann C, Berg D, Bauer P, et al Extended mutation analysis and association studies of Nurr1 (NR4A2) in Parkinson disease. Neurology. 2004; 62: 1231–1232. 15079038 10.1212/01.wnl.0000118285.18383.90

[9] Tan EK, Chung H, Zhao Y, Shen H, Chandran VR, Tan C, et al Genetic analysis of Nurr1 haplotypes in Parkinson's disease. Neurosci Lett. 2003; 347: 139–142. 12875905 10.1016/s0304-3940(03)00539-1

[10] Chu Y, Kompoliti K, Cochran EJ, Mufson EJ, Kordower JH. Age-related decreases in Nurr1 immunoreactivity in the human substantia nigra. J Comp Neurol. 2002; 450: 203–214. 12209851 10.1002/cne.10261

[11] Moran LB, Croisier E, Duke DC, Kalaitzakis ME, Roncaroli F, Deprez M, et al Analysis of alpha-synuclein, dopamine and parkin pathways in neuropathologically confirmed parkinsonian nigra. Acta Neuropathol. 2007; 113: 253–263. 17203291 10.1007/s00401-006-0181-6

[12] Chu Y, Le W, Kompoliti K, Jankovic J, Mufson EJ, Kordower JH. Nurr1 in Parkinson's disease and related disorders. J Comp Neurol. 2006; 494: 495–514. 16320253 10.1002/cne.20828 PMC2564615

[13] Zhang L, Le W, Xie W, Dani JA. Age-related changes in dopamine signaling in Nurr1 deficient mice as a model of Parkinson's disease. Neurobiol Aging. 2012; 33: 1001 e1007–1016. 10.1016/j.neurobiolaging.2011.03.022 PMC3155628 21531044

[14] Kadkhodaei B, Alvarsson A, Schintu N, Ramskold D, Volakakis N, Joodmardi E, et al Transcription factor Nurr1 maintains fiber integrity and nuclear-encoded mitochondrial gene expression in dopamine neurons. Proc Natl Acad Sci U S A. 2013; 110: 2360–2365. 10.1073/pnas.1221077110 23341612 PMC3568335

[15] Zetterstrom RH, Solomin L, Jansson L, Hoffer BJ, Olson L, Perlmann T. Dopamine neuron agenesis in Nurr1-deficient mice. Science. 1997; 276: 248–250. 9092472 10.1126/science.276.5310.248

[16] Decressac M, Kadkhodaei B, Mattsson B, Laguna A, Perlmann T, Bjorklund A. alpha-Synuclein-induced down-regulation of Nurr1 disrupts GDNF signaling in nigral dopamine neurons. Sci Transl Med. 2012; 4: 163ra156 10.1126/scitranslmed.3004676 23220632

[17] Volpicelli F, Caiazzo M, Greco D, Consales C, Leone L, Perrone-Capano C, et al Bdnf gene is a downstream target of Nurr1 transcription factor in rat midbrain neurons in vitro. J Neurochem. 2007; 102: 441–453. 17506860 10.1111/j.1471-4159.2007.04494.x

[18] Saijo K, Winner B, Carson CT, Collier JG, Boyer L, Rosenfeld MG, et al A Nurr1/CoREST pathway in microglia and astrocytes protects dopaminergic neurons from inflammation-induced death. Cell. 2009; 137: 47–59. 10.1016/j.cell.2009.01.038 19345186 PMC2754279

[19] Deleidi M, Isacson O. Viral and inflammatory triggers of neurodegenerative diseases. Sci Transl Med. 2012; 4: 121ps123 10.1126/scitranslmed.3003492 22344685 PMC3982831

[20] Deleidi M, Hallett PJ, Koprich JB, Chung CY, Isacson O. The Toll-like receptor-3 agonist polyinosinic:polycytidylic acid triggers nigrostriatal dopaminergic degeneration. J Neurosci. 2010; 30: 16091–16101. 10.1523/JNEUROSCI.2400-10.2010 21123556 PMC3075577

[21] Koprich JB, Reske-Nielsen C, Mithal P, Isacson O. Neuroinflammation mediated by IL-1beta increases susceptibility of dopamine neurons to degeneration in an animal model of Parkinson's disease. J Neuroinflammation. 2008; 5: 8 10.1186/1742-2094-5-8 18304357 PMC2292163

[22] Decressac M, Volakakis N, Bjorklund A, Perlmann T. NURR1 in Parkinson disease—from pathogenesis to therapeutic potential. Nat Rev Neurol. 2013; 9: 629–636. 10.1038/nrneurol.2013.209 24126627

[23] Yi SH, He XB, Rhee YH, Park CH, Takizawa T, Nakashima K, et al Foxa2 acts as a co-activator potentiating expression of the Nurr1-induced DA phenotype via epigenetic regulation. Development. 2014; 141: 761–772. 10.1242/dev.095802 24496614

[24] Couch Y, Alvarez-Erviti L, Sibson NR, Wood MJ, Anthony DC. The acute inflammatory response to intranigral alpha-synuclein differs significantly from intranigral lipopolysaccharide and is exacerbated by peripheral inflammation. J Neuroinflammation. 2011; 8: 166 10.1186/1742-2094-8-166 22122884 PMC3239418

[25] Frank-Cannon TC, Tran T, Ruhn KA, Martinez TN, Hong J, Marvin M, et al Parkin deficiency increases vulnerability to inflammation-related nigral degeneration. J Neurosci. 2008; 28: 10825–10834. 10.1523/JNEUROSCI.3001-08.2008 18945890 PMC2603252

[26] Nguyen TA, Frank-Cannon T, Martinez TN, Ruhn KA, Marvin M, Casey B, et al Analysis of inflammation-related nigral degeneration and locomotor function in DJ-1(-/-) mice. J Neuroinflammation. 2013; 10: 50 10.1186/1742-2094-10-50 23622116 PMC3769147

[27] Gao HM, Hong JS, Zhang W, Liu B. Synergistic dopaminergic neurotoxicity of the pesticide rotenone and inflammogen lipopolysaccharide: relevance to the etiology of Parkinson's disease. J Neurosci. 2003; 23: 1228–1236. 12598611 10.1523/JNEUROSCI.23-04-01228.2003 PMC6742266

[28] Mangano EN, Hayley S. Inflammatory priming of the substantia nigra influences the impact of later paraquat exposure: Neuroimmune sensitization of neurodegeneration. Neurobiol Aging. 2009; 30: 1361–1378. 10.1016/j.neurobiolaging.2007.11.020 18187236

[29] Galleguillos D, Fuentealba JA, Gomez LM, Saver M, Gomez A, Nash K, et al Nurr1 regulates RET expression in dopamine neurons of adult rat midbrain. J Neurochem. 2010; 114: 1158–1167. 10.1111/j.1471-4159.2010.06841.x 20533997

[30] Wallen AA, Castro DS, Zetterstrom RH, Karlen M, Olson L, Ericson J, et al Orphan nuclear receptor Nurr1 is essential for Ret expression in midbrain dopamine neurons and in the brain stem. Mol Cell Neurosci. 2001; 18: 649–663. 11749040 10.1006/mcne.2001.1057

[31] Cao JP, Li FZ, Zhu YY, Yuan HH, Yu ZQ, Gao DS. Expressions and possible roles of GDNF receptors in the developing dopaminergic neurons. Brain Res Bull. 2010; 83: 321–330. 10.1016/j.brainresbull.2010.09.009 20884338

[32] Pratt L, Ni L, Ponzio NM, Jonakait GM. Maternal inflammation promotes fetal microglial activation and increased cholinergic expression in the fetal basal forebrain: role of interleukin-6. Pediatr Res. 2013; 74: 393–401. 10.1038/pr.2013.126 23877071

[33] Field R, Campion S, Warren C, Murray C, Cunningham C. Systemic challenge with the TLR3 agonist poly I:C induces amplified IFNalpha/beta and IL-1beta responses in the diseased brain and exacerbates chronic neurodegeneration. Brain Behav Immun. 2010; 24: 996–1007. 10.1016/j.bbi.2010.04.004 20399848 PMC3334265

[34] Konat GW, Borysiewicz E, Fil D, James I. Peripheral challenge with double-stranded RNA elicits global up-regulation of cytokine gene expression in the brain. J Neurosci Res. 2009; 87: 1381–1388. 10.1002/jnr.21958 19115408

[35] Gandhi R, Hayley S, Gibb J, Merali Z, Anisman H. Influence of poly I:C on sickness behaviors, plasma cytokines, corticosterone and central monoamine activity: moderation by social stressors. Brain Behav Immun. 2007; 21: 477–489. 17267173 10.1016/j.bbi.2006.12.005

[36] Hirsch EC, Hunot S. Neuroinflammation in Parkinson's disease: a target for neuroprotection? Lancet Neurol. 2009; 8: 382–397. 10.1016/S1474-4422(09)70062-6 19296921

[37] More SV, Kumar H, Kim IS, Song SY, Choi DK. Cellular and molecular mediators of neuroinflammation in the pathogenesis of Parkinson's disease. Mediators Inflamm. 2013; 2013: 952375 10.1155/2013/952375 23935251 PMC3712244

[38] Imamura K, Hishikawa N, Sawada M, Nagatsu T, Yoshida M, Hashizume Y. Distribution of major histocompatibility complex class II-positive microglia and cytokine profile of Parkinson's disease brains. Acta Neuropathol. 2003; 106: 518–526. 14513261 10.1007/s00401-003-0766-2

[39] Pan J, Zhao YX, Wang ZQ, Jin L, Sun ZK, Chen SD. Expression of FasL and its interaction with Fas are mediated by c-Jun N-terminal kinase (JNK) pathway in 6-OHDA-induced rat model of Parkinson disease. Neurosci Lett. 2007; 428: 82–87. 17959308 10.1016/j.neulet.2007.09.032

[40] Hayley S, Crocker SJ, Smith PD, Shree T, Jackson-Lewis V, Przedborski S, et al Regulation of dopaminergic loss by Fas in a 1-methyl-4-phenyl-1,2,3,6-tetrahydropyridine model of Parkinson's disease. J Neurosci. 2004; 24: 2045–2053. 14985447 10.1523/JNEUROSCI.4564-03.2004 PMC6730390

[41] Woodcock T, Morganti-Kossmann MC. The role of markers of inflammation in traumatic brain injury. Front Neurol. 2013; 4: 18 10.3389/fneur.2013.00018 23459929 PMC3586682

[42] Helmy AA, Naseer MM, Shafie SE, Nada MA. Role of interleukin 6 and alpha-globulins in differentiating Alzheimer and vascular dementias. Neurodegener Dis. 2012; 9: 81–86. 10.1159/000329568 22133543

[43] Silvestroni A, Faull RL, Strand AD, Moller T. Distinct neuroinflammatory profile in post-mortem human Huntington's disease. Neuroreport. 2009; 20: 1098–1103. 10.1097/WNR.0b013e32832e34ee 19590393

[44] Chung CY, Koprich JB, Siddiqi H, Isacson O. Dynamic changes in presynaptic and axonal transport proteins combined with striatal neuroinflammation precede dopaminergic neuronal loss in a rat model of AAV alpha-synucleinopathy. J Neurosci. 2009; 29: 3365–3373. 10.1523/JNEUROSCI.5427-08.2009 19295143 PMC2693917

[45] Gillardon F, Schmid R, Draheim H. Parkinson's disease-linked leucine-rich repeat kinase 2(R1441G) mutation increases proinflammatory cytokine release from activated primary microglial cells and resultant neurotoxicity. Neuroscience. 2012; 208: 41–48. 10.1016/j.neuroscience.2012.02.001 22342962

[46] Daher JP, Volpicelli-Daley LA, Blackburn JP, Moehle MS, West AB. Abrogation of alpha-synuclein-mediated dopaminergic neurodegeneration in LRRK2-deficient rats. Proc Natl Acad Sci U S A. 2014; 111: 9289–9294. 10.1073/pnas.1403215111 24927544 PMC4078806

